# Draft genome sequence of *Enterobacter* sp. LM3, isolated from activated sludge

**DOI:** 10.1128/mra.00140-25

**Published:** 2025-07-31

**Authors:** Dan Wu, Lina Kong, Yang Wang, Yongjian Piao, Yixing Yuan, Fugui Zhang, Lixia Zhang, Shuang Zhang

**Affiliations:** 1 Longjiang Environmental Protection Group Co., Ltd, Harbin, China; 2 School of Environment, Harbin Institute of Technology 47822, Harbin, China; Portland State University, Portland, Orlando, USA

**Keywords:** draft genome sequence, *Enterobacter*, sewage treatment, cold tolerance

## Abstract

We report the draft genome of *Enterobacter* sp. LM3, isolated from the activated sludge of a sewage treatment plant. The draft genome of strain LM3 is 4,376,652 bp, with a GC content of 53.5% and 4,042 protein-coding genes.

## ANNOUNCEMENT

Some species of the genus *Enterobacter* achieved heterotrophic nitrification and aerobic denitrification processes (1–3). However, the ability to perform denitrification under low-temperature conditions was rarely reported (4). In this study, activated sludge samples were collected from Harbin Wastewater Treatment Plant, Heilongjiang Province, China, in the winter of 2023. A 10 mL sludge sample was transferred to 90 mL of denitrification broth medium (5) and incubated at 15°C for 5 days. Thereafter, the enriched solution underwent serial dilution. The diluted sample was spread onto the BTB medium plates (6). After repeated separation and purification, a strain designated as LM3 was picked out. When strain LM3 was cultured on BTB medium at 15°C, it caused the medium around the colony to turn blue, which suggests its ability to perform denitrification under low-temperature conditions (6).

Genomic DNA was isolated from a monoculture of strain LM3 cultivated at 15°C in denitrification broth medium for 5 days using the Bacteria DNA Kit (OMEGA). The 16S rRNA gene analysis of LM3 was conducted according to the method described by Zhao et al (7). Results obtained using BLAST (8) in NCBI (https://www.ncbi.nlm.nih.gov/) indicated that the 16S rRNA gene of LM3 exhibited 99.72% sequence identity to *Enterobacter cloacae* AP022133. Highly purified DNA sample (OD260/280 = 1.8–2.0, >6 µg) was utilized to construct the DNA library by the TruSeq Nano DNA Sample Prep Kit (Illumina). The draft genome was then sequenced using the Illumina NovaSeq 6000 (PE150), generating 2,831,368 raw paired-end reads. The reads were trimmed and quality controlled by Trimmomatic v0.36 (9) (ILLUMINACLIP:Adapter:fasta:2:30:10, SLIDINGWINDOW:4:15, MINLEN:75). The genome assembly was performed using ABySS v2.2.0 (10) with multiple-Kmer parameters. The completeness for the assembly was estimated by BUSCO v5.3.2 (11). Coding genes of the sequenced genome were annotated using GeneMarkS v0.2.11 (12) (-gcode 11 --prok) and PGAP v6.9 (13). All gene models were blastp against databases such as Kyoto Encyclopedia of Genes and Genomes (KEGG) (https://www.genome.jp/kegg/), Gene Ontology (GO) (http://www.geneontology.org/), etc., to obtain annotation information. KEGG was compared by KofamScan v1.2.0 (14), GO was compared by eggNOG-mapper v5.0 (15), tRNAs were determined by tRNAscan-SE v2.0.4 (16), and rRNAs were predicted by RNAmmer v1.2 (17). Average Nucleotide Identity (ANI) was analyzed by Pyani v0.2.11 (18). Default parameters were used except where otherwise noted.

The assembled genome of strain LM3 contains 64 scaffolds, with 200× coverage and an N50 value of 199,241 bp. The draft genome is composed of 4,376,652 bp, with a G+C content of 53.5%. The completeness for the assembly was 97.6%. A total of 4,042 putative protein-coding genes, along with 68 tRNA and 2 rRNA elements, were predicted. We annotated genes responsible for cold resistance, such as ATP-dependent RNA helicase DeaD, trehalose 6-phosphate synthase, and regulatory DnaK co-chaperone (19, 20). Genes related to denitrification were annotated, such as nitrate reductase alpha/beta/gamma subunit, nitrite reductase large/small subunit, and nitrate/nitrite transporter (21). The result of ANI analysis (22) showed the best matching strain of LM3 is *Enterobacter mori* CP061801 with the ANI values of 99.5%, indicating that strain LM3 belongs to the genus *Enterobacter*.

## Data Availability

This whole-genome shotgun project has been deposited at DDBJ/ENA/GenBank under the accession number JBJURN000000000 (BioProject number PRJNA1193794). Raw sequences were deposited in the Sequence Read Archive (SRA) database under the accession number SRR31605496.
